# Author Correction: Reversal of pre-existing NGFR-driven tumor and immune therapy resistance

**DOI:** 10.1038/s41467-023-35852-2

**Published:** 2023-02-07

**Authors:** Julia Boshuizen, David W. Vredevoogd, Oscar Krijgsman, Maarten A. Ligtenberg, Stephanie Blankenstein, Beaunelle de Bruijn, Dennie T. Frederick, Juliana C. N. Kenski, Mara Parren, Marieke Brüggemann, Max F. Madu, Elisa A. Rozeman, Ji-Ying Song, Hugo M. Horlings, Christian U. Blank, Alexander C. J. van Akkooi, Keith T. Flaherty, Genevieve M. Boland, Daniel S. Peeper

**Affiliations:** 1 Division of Molecular Oncology and Immunology, Oncode Institute, The Netherlands Cancer Institute, Amsterdam, The Netherlands; 2 Division of Surgical Oncology, The Netherlands Cancer Institute, Amsterdam, The Netherlands; 3 Department of Surgical Oncology, Massachusetts General Hospital, Boston, MA USA; 4 Division of Animal Pathology, The Netherlands Cancer Institute, Amsterdam, The Netherlands; 5 Division of Pathology, The Netherlands Cancer Institute, Amsterdam, The Netherlands; 6 Department of Medical Oncology, Massachusetts General Hospital, Boston, MA USA

Correction to: *Nature Communications* 10.1038/s41467-020-17739-8, published online 07 August 2020

The original version of this Article contained an error in Fig. 5e, in which the bottom 25% (NGFR^lo^) and top 25% (NGFR^hi^) groups were reversed. The correct version of Fig. 5 is:
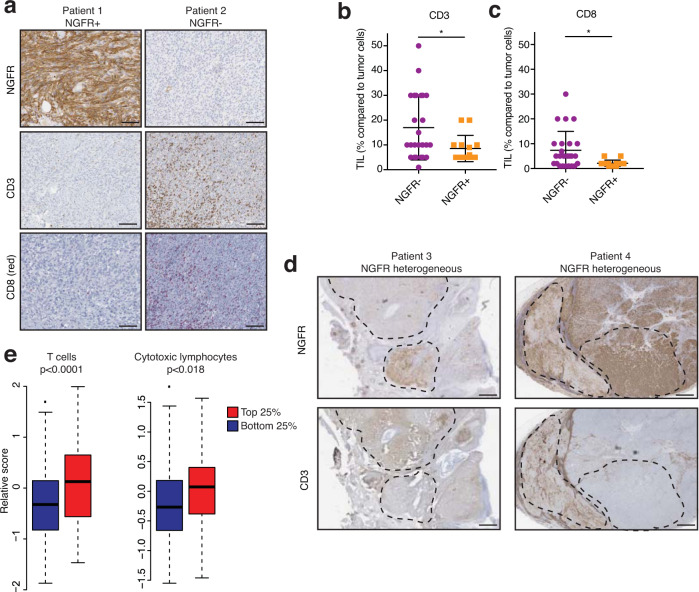


which replaces the previous incorrect version:
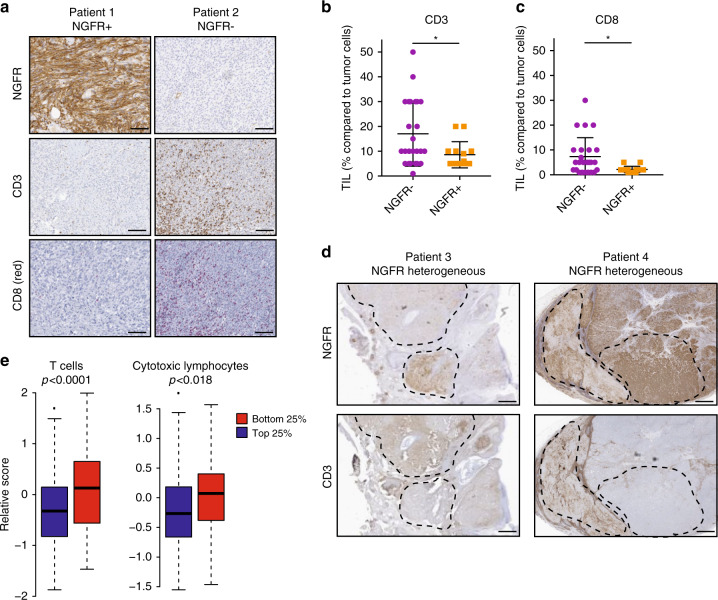


As a consequence, the original Article contains an error in the text describing Fig. 5e which incorrectly reads ‘These findings were confirmed by performing a computational estimation of T cell infiltrates in NGFR^lo^ vs NGFR^hi^ melanoma tumors in TCGA using MCP counter (Fig. 5e)’. This should read ‘Computational estimation of T cell infiltrates in NGFR^lo^ vs NGFR^hi^ melanomas in TCGA using MCP counter showed an opposite pattern (Fig. 5e). This apparent discrepancy between bulk RNA sequencing and immunohistochemistry analyses underscores the importance of including spatial information (Fig. 5a–d) for complete interpretation of these types of analyses, as also others have noted^19,35^. This association between T cells and NGFR^hi^ melanoma cells may be caused by the initial requirement of T cells to establish an NGFR^hi^, T cell-resistant (TR) tumor population before being excluded by as yet to be identified mechanisms.’, where correct text replaces incorrect.

The original version of this Article omitted the details of the analysis of Fig. 5e in the ‘Patient RNA sequencing datasets’ section in the Methods:

‘RNA expression data for melanoma (SKCM, *n* = 472) samples were downloaded from the TCGA portal using the R-package ‘TCGAbiolinks’. The downloaded count data was normalized using DESeq2 (Ref. ^45^) and the Z-score calculated (number of standard deviations below or above the population mean).

The tool ‘MCPcounter’ (https://github.com/ebecht/MCPcounter) was used to estimate the abundance of immune and stromal cells in the downloaded TCGA samples. MCPcounter was run on the 472 TCGA melanoma samples for all gene signatures as provided by MCPcounter (http://raw.githubusercontent.com/ebecht/MCPcounter/master/Signatures/genes.txt).

For the gene signatures “T cells” and “Cytotoxic lymphocytes”, boxplots were made based on the top 25% highest (*n* = 118) and 25% lowest (*n* = 118) NGFR-expressing samples. *P*-values were based on an unpaired t-test. The code used is available on GitHub https://github.com/PeeperLab/NGFR_NatComm2020’

New references have been added as a consequence of this correction:

(19) Landsberg, J. et al. Melanomas resist T-cell therapy through inflammation-induced reversible dedifferentiation. *Nature* **490**, 412–416 (2012).

(35) Liu, D. et al. Evolution of delayed resistance to immunotherapy in a melanoma responder. *Nat Med.* 1–8 10.1038/s41591-021-01331-8 (2021).

(45) Love, M. I., Huber, W. & Anders, S. Moderated estimation of fold change and dispersion for RNA-seq data with DESeq2. *Genome Biol.* **15**, 550 (2014).

This has been corrected in both the PDF and HTML versions of the Article.

