# Supplementary material for: Reversal of pre-existing NGFR-driven tumor and immune therapy resistance
Source: Nat Commun. 2020 Aug 7;11:3946. doi: 10.1038/s41467-020-17739-8 (PMC7414147; doi:10.1038/s41467-020-17739-8)
Supplement: Supplementary file 1 — Supplementary Information [file 41467_2020_17739_MOESM1_ESM.pdf]

Supplementary Figure 1

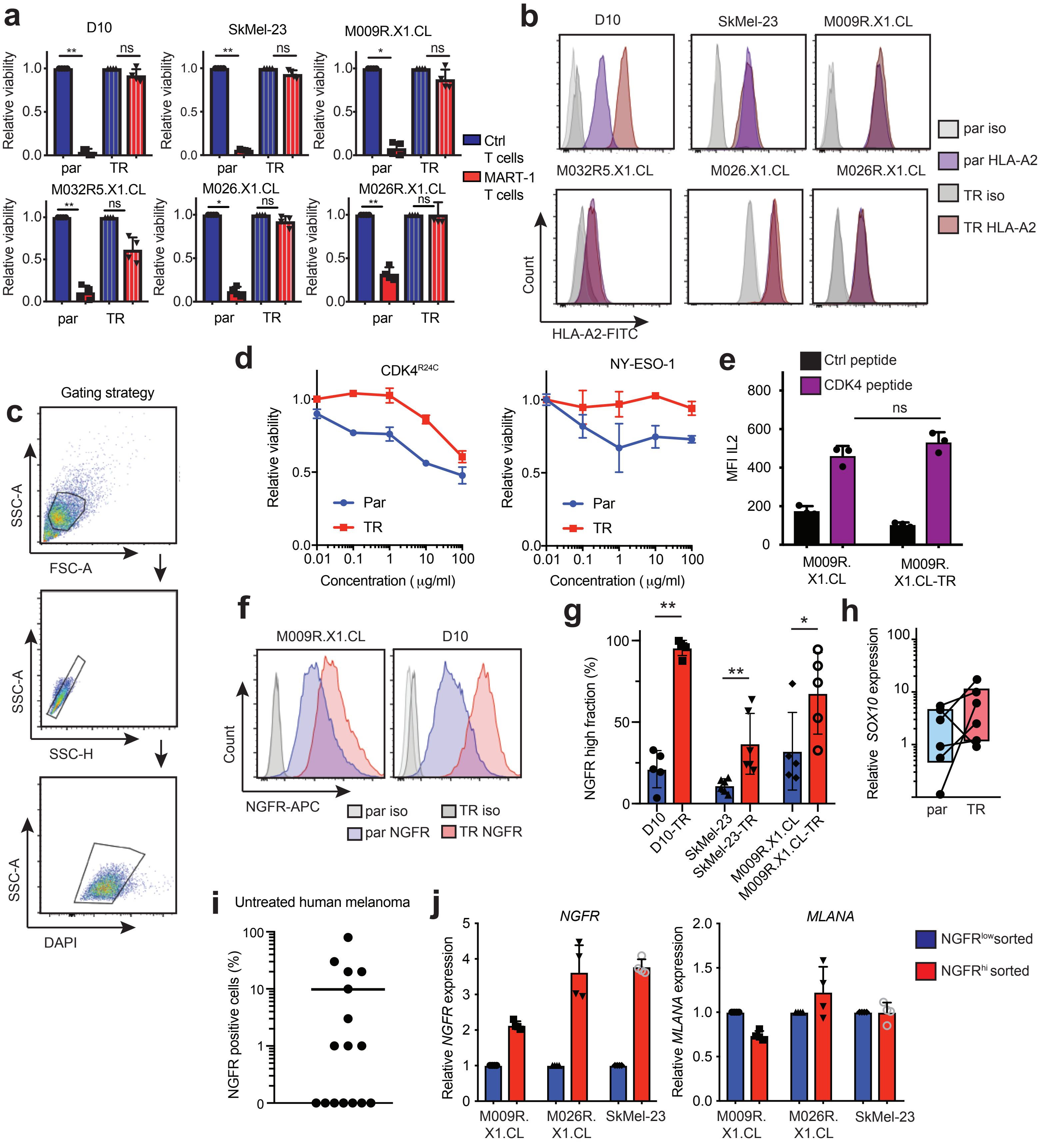

Supplementary Figure 1. Melanoma fractions resistant to antigen-specific T cells. a) Quantification of colony formation assays of indicated parental cell lines and their T cell- resistant (TR) counterparts, treated with control (Ctrl) or MART-1 T cells for 24 hours in a 1:1 ratio. Afterwards, T cells were washed away and plates were stained with crystal violet after 3 days and quantified. Error bars represent S.D. of four independent replicates. Statistical analysis by Mann-Whitney test; * $p<0.05$, ** $p<0.01$, ns = not significant. (b) Flow cytometry of HLA-A2 expression in matched cell lines. (c) Gating strategy of flow cytometry experiments. (d) Quantification of cell viability after T cell attack in D10 cells using two different TCRs (CDKR24C and NY-ESO-1 reactive). Cells were loaded with respective peptides for 3 hours at room temperature and sequentially subjected to T cells for 24 hours. Error bars represent S.D. of three technical replicates of one experiment (which was repeated in two biological replicates and can be found in Source data). (e) IL-2 abundance measured by flow cytometry in supernatant of experiment in Fig. 1e. Error bars represent S.D. of three independent replicates. Statistical testing by Mann-Whitney, ns = not significant. (f) Flow cytometry of NGFR expression in matched cell lines. (g) Flow cytometry of NGFR high fractions in matched cell lines. Pooled data of five or six (SkMel-23) independent replicates. Statistical analysis by Mann-Whitney test; * $p<0.05$, ** $p<0.01$. (h) mRNA expression levels of SOX10 in parental versus TR cell lines. Pooled data of six independent cell line pairs, lines indicate each paired parental and TR cell line. One experiment is shown; the data is produced in two independent replicates (which can be found in Source data). (i) Immunohistochemistry quantification of NGFR expression on human melanoma. (j) mRNA expression levels of MLANA and NGFR in sorted cell lines, seven days after sorting. Error bars represent S.D. of four independent replicates. Source data are provided as a Source Data file.

Supplementary Figure 2

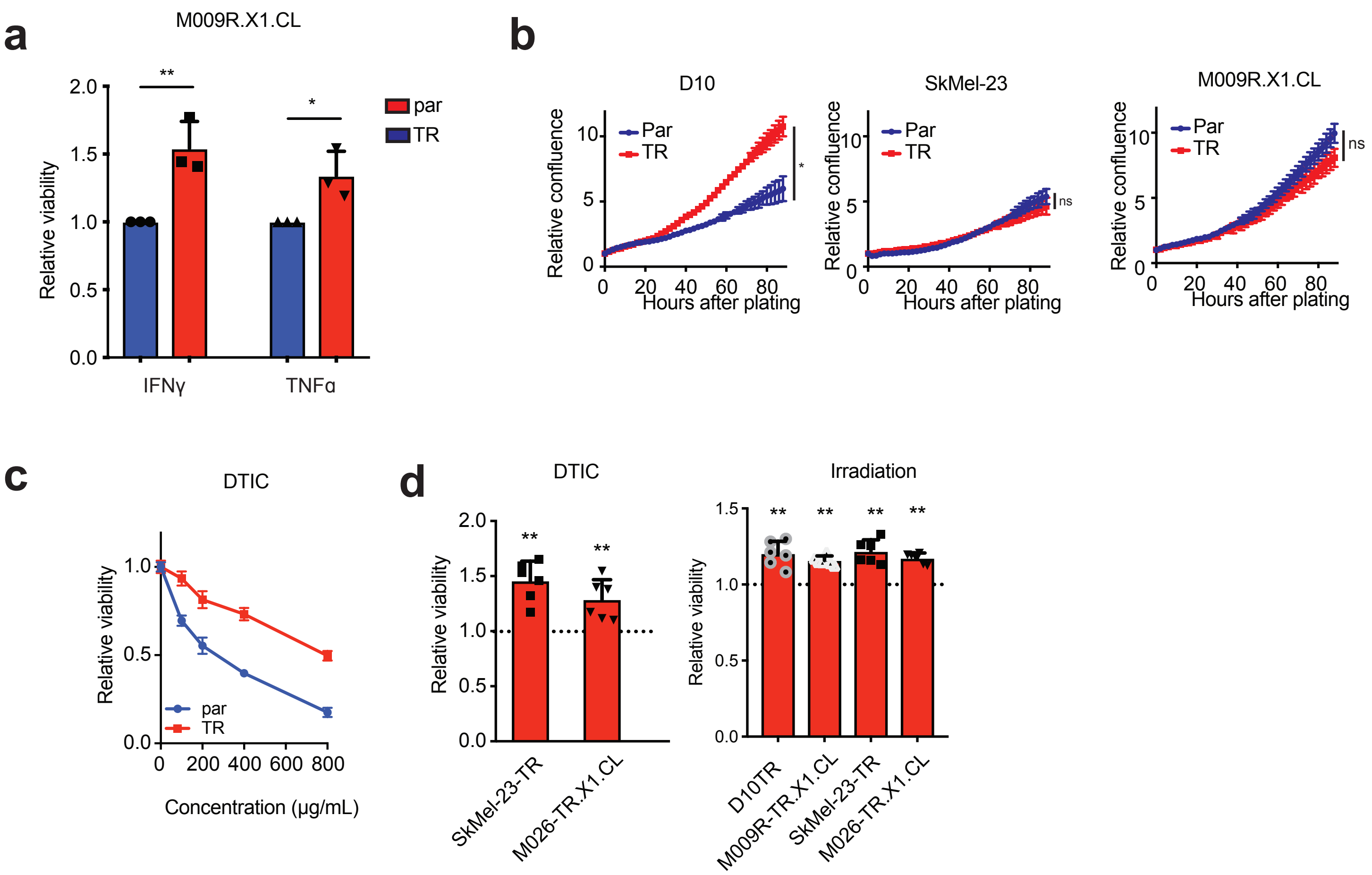

Supplementary Figure 2. NGFR^{hi} melanomas are resistant to multiple therapies. (a) Quantification of colony formation assays of M009R.X1.CL parental cell line and their T cell-resistant (TR) counterpart, treated with indicated cytokines for seven days. Medium was refreshed on day 4. Error bars represent S.D. Statistical analysis by unpaired t-test; ** p<0.01, * p<0.05. (b) Proliferation speed of three parental and TR cell lines. Error bars represent S.D. of three technical replicates; experiment was performed in two independent replicates (which can be found in Source data). Statistical analysis by Mann-Whitney test; * p<0.05, ns = not significant. (c) Cytotoxicity assay in SkMel-23 parental versus TR cells for DTIC (dacarbazine). Error bars represent S.D. of three technical replicates. Quantification in Supplementary Fig 2d. (d) Relative viability of TR cells compared to their parental counterparts at a concentration of 200 µg/ml DTIC or 4 Gy of irradiation. Error bars represent S.D. of three independent replicates with two technical replicates. Statistical analysis by Mann-Whitney test; ** p<0.01. Source data are provided as a Source Data file.

Supplementary Figure 3

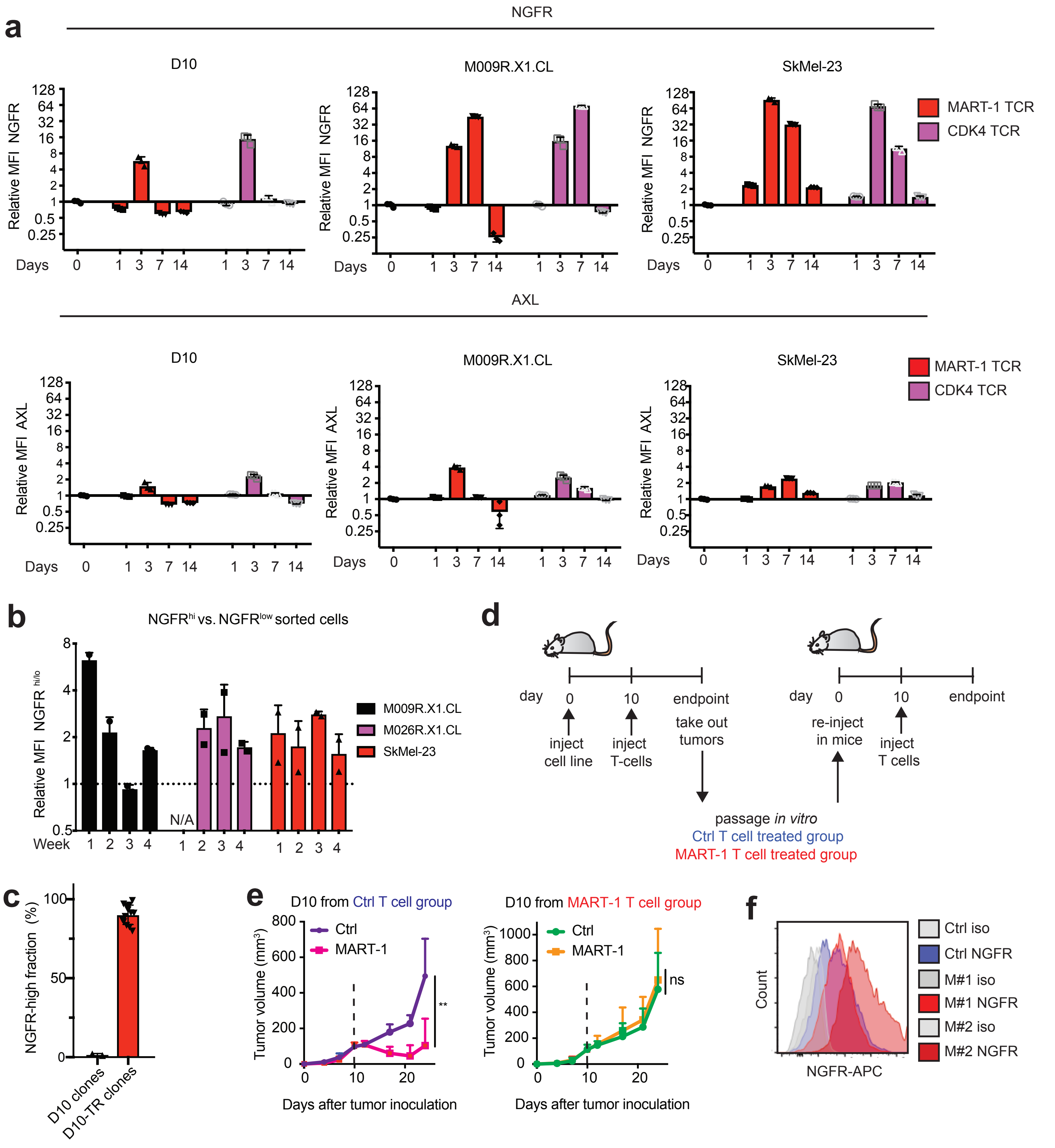

Supplementary Figure 3. Pre-existing NGFR^{hi} cells display a stable phenotype. (a) Relative MFI of NGFR and AXL as assessed by flow cytometry for parental cell lines after a 24 hour co-culture with MART-1 T cells in a 1:8 ratio. After 24 hours, T cells were washed away and the medium was refreshed every 3 days. Cells were subjected to flow cytometry at the indicated timepoints. Error bars represent S.D. of three independent experiments. (b) Relative MFI of NGFR as assessed by flow cytometry for sorted NGFRhi versus NGFRlow cells, after the indicated time since sorting (1-4 weeks). Error bars represent S.D. from two independent replicates. (c) Fraction of NGFR-high tumor cells as assessed by flow cytometry, in D10 parental and TR clones. Average of two independent replicates. (d) Graphic overview of serial transplantations of D10 tumor cells. First, in vivo D10 tumors were subjected to either Ctrl or MART-1 T cells, and tumors were harvested at endpoint (when they reached 1000 mm3), single cell-dissociated and passaged for 3-4 passages in vitro. After expansion, they were injected into a new next cohort of mice, which were sequentially treated with either Ctrl or MART-1 T cells. (e) Comparison of MART-1 T cell response in D10 tumors that were derived from a previous group of Ctrl T cell treatment, versus D10 tumors that were derived from cells that had acquired resistance to MART-1 T cells. All n=7 per group except for D10 Ctrl, which was n=5. Error bars represent S.D. Statistical analysis by Kruskal-Wallis test; * p<0.05, ns = not significant. (f) Flow cytometry of NGFR expression in indicated cell lines. D10 M#1 and M#2 cell lines were both derived from a previously MART-1 T cell treated mouse; Ctrl cell line was derived from a Ctrl T cell treated mouse. Source data are provided as a Source Data file.

Supplementary Figure 4

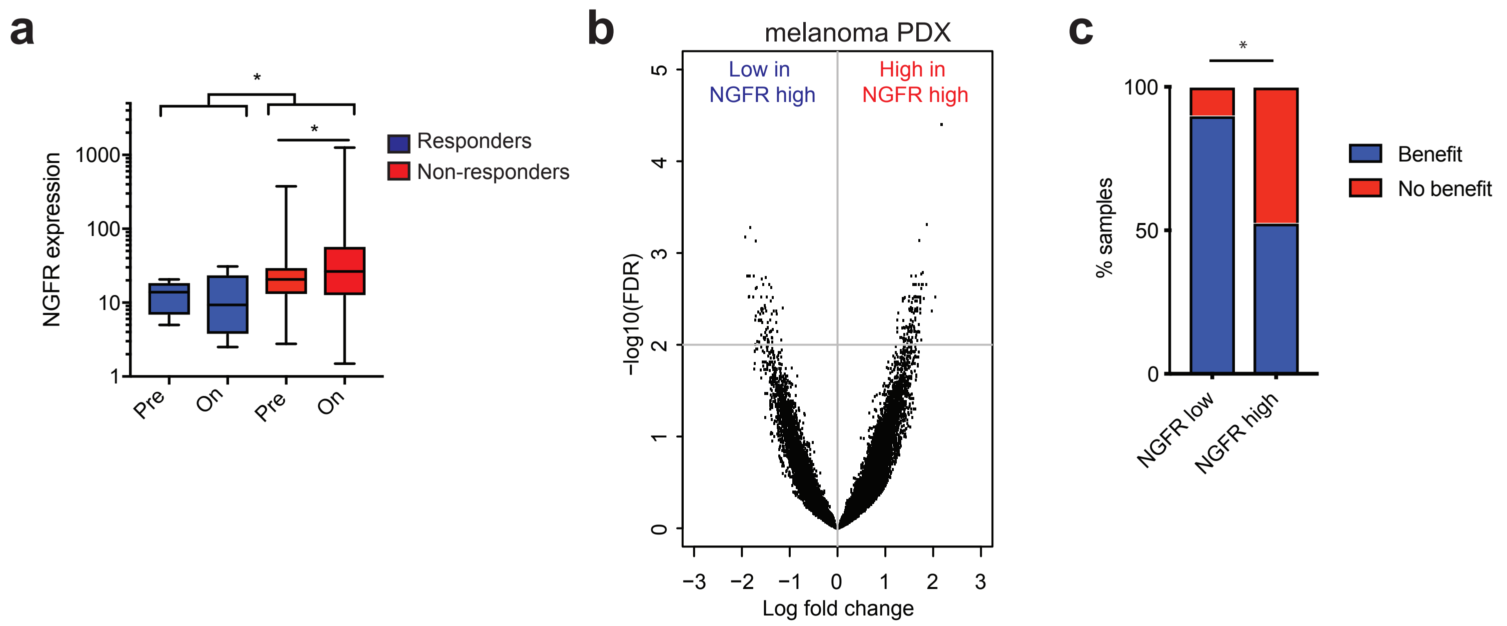

Supplementary Figure 4. NGFR predicts immunotherapy resistance in melanoma patients. (a) NGFR expression levels in indicated samples. Error bars represent S.D. Statistical analysis by Mann-Whitney test. *p<0.05. (b) Differential gene expression in melanoma PDX comparing NGFRhi vs. NGFRlow tumor samples. Genes >-log10(FDR)2 were considered significantly differentially expressed (grey line). These genes were used to create the tumor-intrinsic NGFR-signature. (c) NGFR immunohistochemistry on a set of patients that had clinical benefit or no clinical benefit to anti-PD1 therapy. Patients were scored for NGFR expression in tumor cells specifically, and were considered positive when at least 10% of tumor cells was positive. Statistical analysis by Chi-squared test; *p<0.05. Source data are provided as a Source Data file.

Supplementary Figure 5

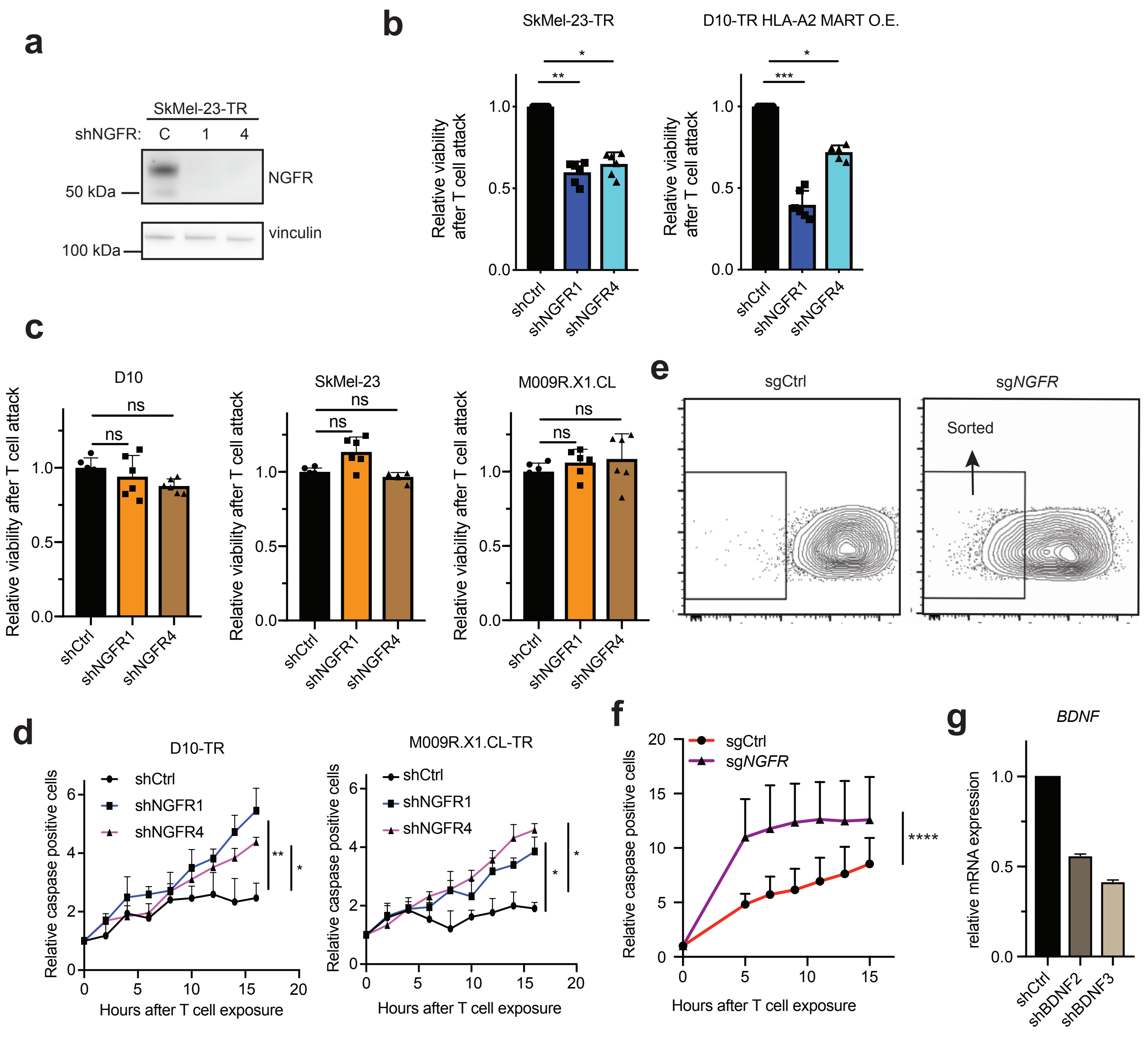

Supplementary Figure 5. Genetic perturbation of NGFR modulates T cell sensitivity. (a) Western Blot analysis of SkMel-23-TR cells after knockdown of NGFR. Vinculin was used as a loading control. One blot of two independent experiments is shown (the other can be found in the Source data). (b) Quantification of MART-1 T cell cytotoxicity in TR cell lines relative to Ctrl T cells (1:1 ratio tumor : T cell) in the presence of absence of shNGFR. Cells were loaded with MART-1 peptide prior to the assay, and cytotoxicity was normalized to shCtrl cell survival after MART-1 T cell attack. Error bars represent S.D. of six independent experiments. Statistical analysis by Mann-Whitney test; ** p<0.01, **** p<0.001. (c) Quantification of MART-1 T cell cytotoxicity in TR cell lines relative to Ctrl T cells (1:1 ratio tumor : T cell) in the presence of absence of shNGFR. Viability was normalized to shCtrl cell survival after MART-1 T cell attack. Error bars represent S.D. of three independent experiments with two replicates each. Statistical analysis by Kruskal-Wallis test; ** p<0.01, **** p<0.001. (d) Quantification of caspase-positive cells after T cell attack in shCtrl and shNGFR cells, as measured by Incucyte. Error bars represent S.D. of three independent replicates. Statistical analysis by Mann-Whitney test, * p<0.05, ** p< 0.01. (e) Flow cytometry-based sort of sgNGFR D10-TR cells, which were then used for Extended Data Fig. 5f. (f) Quantification of caspase-positive cells after T cell attack in sgCtrl or sgNGFR cells (sorted for purity as in panel E). Error bars represent S.D. from three independent replicates. Statistical analysis by Mann-Whitney test; **** p<0.0001. (g) mRNA levels of BDNF in M009R.X1.CL-TR cells expressing either shCtrl or shBDNF. Pooled data of two independent replicates. Source data are provided as a Source Data file.

Supplementary Figure 6

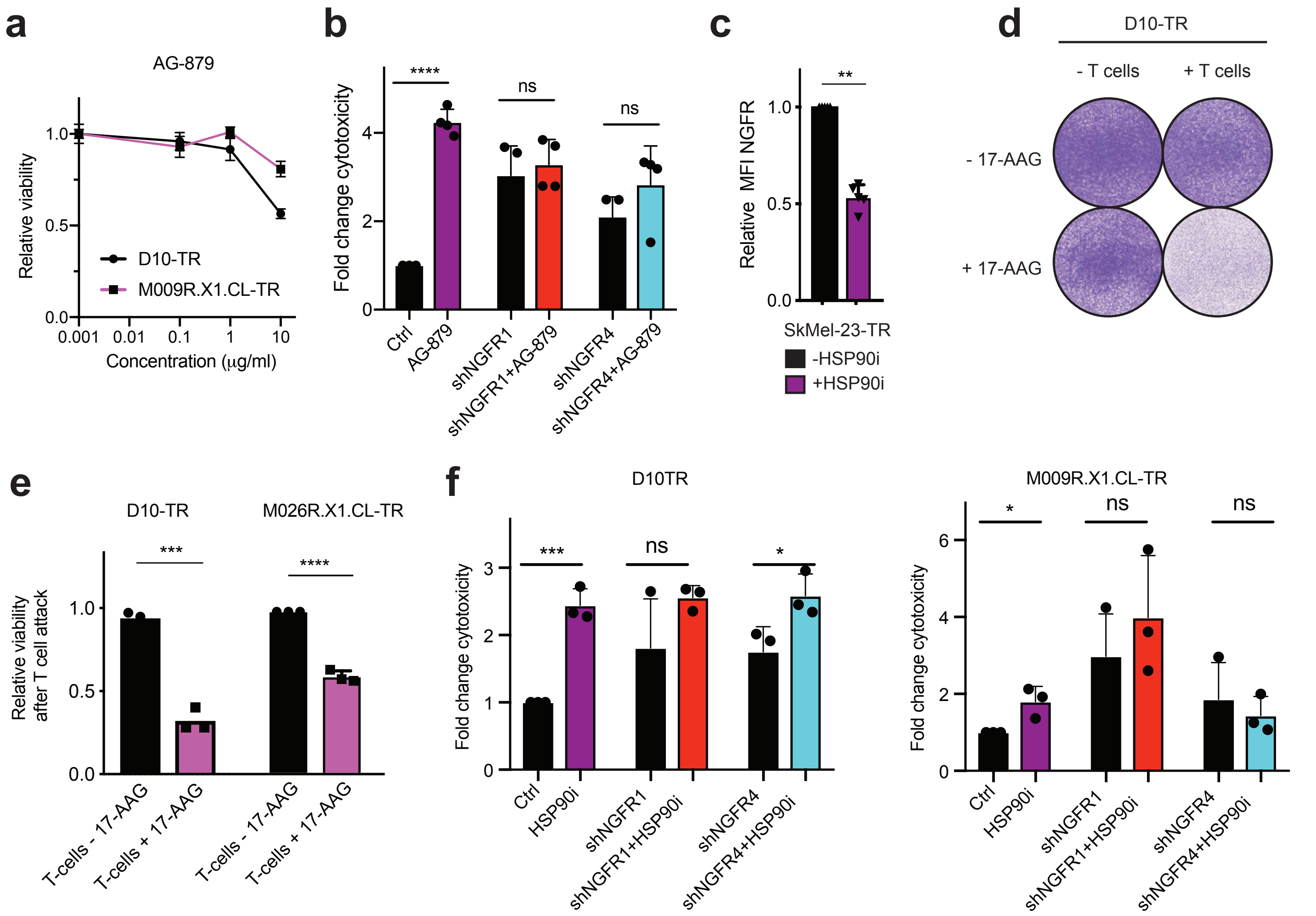

Supplementary Figure 6. Pharmacological reversal of the NGFRhi state restores T cell sensitivity. (a) Viability of TR cells upon AG-879 treatment. Cells were treated for three days. Error bars represent S.D. of three independent replicates. (b) Quantification of MART-1 T cell cytotoxicity in TR cell lines relative to T cell-mediated killing alone (1:1 ratio tumor : T cell) in the presence of absence of shNGFR and presence or absence of AG-879. Cells were loaded with MART-1 peptide prior to the assay, and cytotoxicity was normalized to shCtrl cell survival after MART-1 T cell attack. Error bars represent S.D. of three independent experiments. Statistical analysis by unpaired t-test; **** p<0.001. (c) Quantification of NGFR expression by flow cytometry after 3 days of 250 μM ganetespib treatment in vitro. Error bars represent S.D. of five independent experiments. Statistical analysis by Mann-Whitney, ** p< 0.01. (d) Colony formation assay of D10-TR cells treated with control (Ctrl) or MART-1 T cells in a 1:1 ratio for 72 hours with or without 0.025 mg/ml 17-AAG. Afterwards, plates were washed and stained with crystal violet. Quantification in Extended Data Fig. 6e. (e) Relative viability of indicated cell lines, calculated based on the additive effect of 17-AAG upon MART-1 T cell killing. Error bars represent S.D. from three independent replicates. Statistical analysis by Mann-Whitney test; *** p<0.001, **** p<0.0001. (f) Quantification of MART-1 T cell cytotoxicity in TR cell lines relative to T cell-mediated killing alone (1:1 ratio tumor : T cell) in the presence of absence of shNGFR and presence or absence of HSP90 inhibitor ganetespib (5 nM). Cells were loaded with MART-1 peptide prior to the assay, and cytotoxicity was normalized to shCtrl cell survival after MART-1 T cell attack. Error bars represent S.D. of three independent experiments. Statistical analysis by unpaired t-test; *** p<0.01, * p<0.05. Source data are provided as a Source Data file.

Supplementary Table 1. Primer sequences used in this study.

Gene	Primer sequence
NGFR	F: TCATCCCTGTCTATTGCTCCA<br>R: TGTTCTGCTTGCAGCTGTTC
SOX10	F: CTTTCTTGTGCTGCATACGG<br>R: AGCTCAGCAAGACGCTGG
MLANA	F: GCTCACTTCATCTATGGTTACCC<br>R: GACTCCCAGGATCACTGTCAG
RPL13	F: GAGACAGTTCTGCTGAAGAACTGAA<br>R: TCCGGACGGGCATGAC
BDNF	F: AGCCTTTTCCTCCTGCTGTG<br>R: CCTGGTGGAACTGGGGGTA
