# Supplementary material for: Reversal of pre-existing NGFR-driven tumor and immune therapy resistance
Source: Nat Commun. 2020 Aug 7;11:3946. doi: 10.1038/s41467-020-17739-8 (PMC7414147; doi:10.1038/s41467-020-17739-8)
Supplement: Supplementary file 3 — Description of Additional Supplementary Information [file 41467_2020_17739_MOESM3_ESM.pdf]

Description of Additional Supplementary Files

File Name: Supplementary Data 1
Description: Quantity and quality control of RNA sequencing.
