# Supplementary material for: Reversal of pre-existing NGFR-driven tumor and immune therapy resistance
Source: Nat Commun. 2020 Aug 7;11:3946. doi: 10.1038/s41467-020-17739-8 (PMC7414147; doi:10.1038/s41467-020-17739-8)
Supplement: Supplementary file 5 — Reporting Summary [file 41467_2020_17739_MOESM5_ESM.pdf]

nature research

Corresponding author(s): D.S. Peeper

Last updated by author(s): 2020/7/2

Reporting Summary

Nature Research wishes to improve the reproducibility of the work that we publish. This form provides structure for consistency and transparency in reporting. For further information on Nature Research policies, seeAuthors & Referees and theEditorial Policy Checklist .

Statistics

For all statistical analyses, confirm that the following items are present in the figure legend, table legend, main text, or Methods section.

n/a	Confirmed	
☐	☒	The exact sample size (*n*) for each experimental group/condition, given as a discrete number and unit of measurement
☐	☒	A statement on whether measurements were taken from distinct samples or whether the same sample was measured repeatedly
☐	☒	The statistical test(s) used AND whether they are one- or two-sided<br>*Only common tests should be described solely by name; describe more complex techniques in the Methods section.*
☐	☒	A description of all covariates tested
☐	☒	A description of any assumptions or corrections, such as tests of normality and adjustment for multiple comparisons
☐	☒	A full description of the statistical parameters including central tendency (e.g. means) or other basic estimates (e.g. regression coefficient) AND variation (e.g. standard deviation) or associated estimates of uncertainty (e.g. confidence intervals)
☐	☒	For null hypothesis testing, the test statistic (e.g. *F*, *t*, *r*) with confidence intervals, effect sizes, degrees of freedom and *P* value noted<br>*Give P values as exact values whenever suitable.*
☒	☐	For Bayesian analysis, information on the choice of priors and Markov chain Monte Carlo settings
☒	☐	For hierarchical and complex designs, identification of the appropriate level for tests and full reporting of outcomes
☒	☐	Estimates of effect sizes (e.g. Cohen's *d*, Pearson's *r*), indicating how they were calculated

Our web collection on statistics for biologists contains articles on many of the points above.

Software and code

Policy information about availability of computer code

Data collection	Indicated in Material & Methods, page 19-26
Data analysis	Indicated in Material & Methods, page 24-26

For manuscripts utilizing custom algorithms or software that are central to the research but not yet described in published literature, software must be made available to editors/reviewers. We strongly encourage code deposition in a community repository (e.g. GitHub). See the Nature Research guidelines for submitting code & software for further information.

Data

Policy information about availability of data

All manuscripts must include a data availability statement. This statement should provide the following information, where applicable:

- Accession codes, unique identifiers, or web links for publicly available datasets
- A list of figures that have associated raw data
- A description of any restrictions on data availability

A data availability statement is made on page 24 of the manucript:
RNA read count data of the melanoma PDX samples were downloaded from GEO (GSE129127). Raw RNA sequence data (fastq files) were downloaded from SRA for the Hugo (PRJNA312948) and Riaz (PRJNA356761) datasets. The TR sequencing dataset is available on GEO (GSE147091). The analysis of Fig. 5e was performed on the TCGA melanoma database. Codes that were used are XenofilteR, HTSeq-count, and DESeq2.The gene lists for the cell states/subtypes were taken from Tsoi et al. All relevant data is also available from the authors. The source data underlying the figures are provided as Source Data files.

Field-specific reporting

Please select the one below that is the best fit for your research. If you are not sure, read the appropriate sections before making your selection.

- [x] Life sciences
- [ ] Behavioural & social sciences
- [ ] Ecological, evolutionary & environmental sciences

For a reference copy of the document with all sections, see nature.com/documents/nr-reporting-summary-flat.pdf

Life sciences study design

All studies must disclose on these points even when the disclosure is negative.

Sample size	Sample size was determined for mouse experiments on a power of 0.8, a 0.05 and estimated effect sizes + standard deviations using the program G*Power.<br>Sample size for in vitro experiments were always performed at least in two independent experiments.
Data exclusions	No data was excluded, except in mouse experiments when animals were experiencing experiment-unrelated health issues.
Replication	Reproducibility was consistent across different biological replicates of experiments.
Randomization	Randomization occurred in a blinded fashion for mouse experiments, when tumors reached an average of 100 mm3.
Blinding	Researchers were blinded to treatments or groups for in vivo experiments; moreover, measurements were performed by an animal technician (and not the primary researcher), in a blinded fashion.

Reporting for specific materials, systems and methods

We require information from authors about some types of materials, experimental systems and methods used in many studies. Here, indicate whether each material, system or method listed is relevant to your study. If you are not sure if a list item applies to your research, read the appropriate section before selecting a response.

Materials & experimental systems

n/a	Involved in the study	
[ ]	[x]	Antibodies
[ ]	[x]	Eukaryotic cell lines
[x]	[ ]	Palaeontology
[ ]	[x]	Animals and other organisms
[ ]	[x]	Human research participants
[x]	[ ]	Clinical data

Methods

n/a	Involved in the study	
[x]	[ ]	ChIP-seq
[ ]	[x]	Flow cytometry
[x]	[ ]	MRI-based neuroimaging

Antibodies

Antibodies used	Primary antibodies for Western Blot:<br>NGFR (1:1000, #8238, CST)<br>vinculin (1:10,000, V9131-100UL, Sigma)<br>GAPDH (1:1000, 1617002D09, Absea)<br>Secondary antibodies: goat anti-rabbit peroxidase conjugate (1:5,000, G21234) and goat anti-mouse (1:5,000, G21040), both Invitrogen<br>Flow cytometry:<br>mouse TCR β chain (BD Pharmingen, 553172)<br>HLA-A2-FITC conjugated antibody (1:50, 551285, BD)<br>NGFR-APC (1:200, 345107,Biolegend)<br>AXL-PE conjugated antibody (1:200, FAB154P, R&D)<br>IHC:<br>NGFR (1:400, 8238, CST)<br>Secondary antibody Polymer-HRP Anti-Rabbit Envision (K4011, Dako)
Validation	NGFR: https://www.cellsignal.com/products/primary-antibodies/p75ntr-d4b3-xp-rabbit-mab/8238<br>Vinculin: https://www.sigmaaldrich.com/catalog/product/sigma/v9131?lang=en&region=NL<br>GAPDH: http://www.absea-antibody.com/Upfiles/201911120513654998.pdf

	TCR-B chain: https://www.bdbiosciences.com/us/reagents/research/antibodies-buffers/immunology-reagents/anti-mouse-antibodies/cell-surface-antigens/pe-hamster-anti-mouse-tcr-chain-h57-597/p/553172<br>HLA-A2: https://www.bdbiosciences.com/us/applications/research/b-cell-research/surface-markers/human/fitc-mouse-anti-human-hla-a2-bb72/p/551285<br>NGFR-APC: https://www.biolegend.com/de-de/products/apc-anti-human-cd271-ngfr-antibody-6877<br>AXL-PE: https://www.rndsystems.com/products/human-axl-pe-conjugated-antibody-108724_fab154p

Eukaryotic cell lines

Policy information about cell lines

Cell line source(s)	All melanoma cell lines were obtained from the Peeper laboratory cell line stock and can be bought from ATCC or are generated in-house (PDX derived cell lines, Kemper et al. 2016)
Authentication	STR profiling was done on all cell lines.
Mycoplasma contamination	Cell lines were tested each month to be mycoplasma negative.
Commonly misidentified lines<br>(See ICLAC register)	None

Animals and other organisms

Policy information about studies involving animals; ARRIVE guidelines recommended for reporting animal research

Laboratory animals	Done (page 21 manuscript)<br>All in vivo experiments were performed in male or female 8-12 week old NSG or NSG-b2Mnull mice (The Jackson Laboratory).
Wild animals	N/A
Field-collected samples	N/A
Ethics oversight	Animal experiments were approved by the animal experimental committee (Instantie voor Dierenwelzijn) of the institute and performed according to Dutch law.

Note that full information on the approval of the study protocol must also be provided in the manuscript.

Human research participants

Policy information about studies involving human research participants

Population characteristics	Untreated melanoma patients with stage I-IV disease. No selection on age, gender or mutations were made. This is a retrospective cohort from patients from our hospital (see also below).
Recruitment	Informed consent was received from all patients for secondary use of tumor tissue. This tissue is derived from standard diagnostic procedures and was utilized for research purposes by informed consent.
Ethics oversight	The collection and use of human tissue was approved by the Internal Review Board of the Antoni van Leeuwenhoek. Informed consent was received from all patients for secondary use of tumor tissue.

Note that full information on the approval of the study protocol must also be provided in the manuscript.

Flow Cytometry

Plots

Confirm that:

☒ The axis labels state the marker and fluorochrome used (e.g. CD4-FITC).

☒ The axis scales are clearly visible. Include numbers along axes only for bottom left plot of group (a 'group' is an analysis of identical markers).

☒ All plots are contour plots with outliers or pseudocolor plots.

☒ A numerical value for number of cells or percentage (with statistics) is provided.

Methodology

Sample preparation	Page 21:<br>Live cells were stained for 30 minutes at 4C.

Instrument	Page 21:<br>LSRII or LSR Fortessa (BD Biosciences). For the NGFR and single-cell sort, melanoma cells were sorted on the FACSAriaII (BD Biosciences).
Software	FlowJo
Cell population abundance	Post-purity sort was performed directly after sorting but also in a weekly fashion afterwards.
Gating strategy	Gating was performed as follows:<br>- SSC-A-FSC-A gating on cells<br>SSC-A/SSC-H gating for single cells<br>DAPI for live cells<br>--> Population of interest.

☒ Tick this box to confirm that a figure exemplifying the gating strategy is provided in the Supplementary Information.
